# Host Immune Responses Differ between *M*. *africanum*- and *M*. *tuberculosis*-Infected Patients following Standard Anti-tuberculosis Treatment

**DOI:** 10.1371/journal.pntd.0004701

**Published:** 2016-05-18

**Authors:** Leopold D. Tientcheu, Mariëlle C. Haks, Schadrac C. Agbla, Jayne S. Sutherland, Ifedayo M. Adetifa, Simon Donkor, Edwin Quinten, Mohammed Daramy, Martin Antonio, Beate Kampmann, Tom H. M. Ottenhoff, Hazel M. Dockrell, Martin O. Ota

**Affiliations:** 1 Vaccines and Immunity Theme, Medical Research Council Unit, The Gambia, Banjul, The Gambia; 2 Department of Immunology and Infection, Faculty of Infectious and Tropical Diseases, London School of Hygiene and Tropical Medicine, London, United Kingdom; 3 Department of Biochemistry, Faculty of Science, University of Yaoundé 1, Yaoundé, Cameroon; 4 Department of Infectious Diseases, Leiden University Medical Center, Leiden, The Netherlands; 5 Department of Medical Statistics, Faculty of Epidemiology and Population Health, London School of Hygiene and Tropical Medicine, London, United Kingdom; 6 Disease Control and Elimination Theme, Medical Research Council Unit, The Gambia, Fajara, The Gambia; 7 Department of Infectious Diseases Epidemiology, Faculty of Epidemiology and Population Health, London School of Hygiene and Tropical Medicine, London, United Kingdom; 8 Department of Pathogen Molecular Biology, Faculty of Infectious and Tropical Diseases, London School of Hygiene & Tropical Medicine, London, United Kingdom; 9 Microbiology and Infection Unit, Warwick Medical School, University of Warwick, Coventry, United Kingdom; 10 World Health Organization Regional Office for Africa, Brazzaville, Congo; University of Tennessee, UNITED STATES

## Abstract

Epidemiological differences exist between *Mycobacterium africanum* (*Maf*)- and *Mycobacterium tuberculosis* (*Mtb*)-infected patients, but to date, contributing host factors have not been characterised. We analysed clinical outcomes, as well as soluble markers and gene expression profiles in unstimulated, and ESAT6/CFP-10-, whole-*Maf*- and *Mtb*-stimulated blood samples of 26 *Maf*- and 49 *Mtb*-HIV-negative tuberculosis patients before, and after 2 and 6 months of anti-tuberculosis therapy. Before treatment, both groups had similar clinical parameters, but differed in few cytokines concentration and gene expression profiles. Following treatment the body mass index, skinfold thickness and chest X-ray scores showed greater improvement in the *Mtb-* compared to *Maf*-infected patients, after adjusting for age, sex and ethnicity (p = 0.02; 0.04 and 0.007, respectively). In addition, in unstimulated blood, IL-12p70, *IL12A* and *TLR9* were significantly higher in *Maf*-infected patients, while IL-15, IL-8 and MIP-1α were higher in *Mtb*-infected patients. Overnight stimulation with ESAT-6/CFP-10 induced significantly higher levels of IFN-γ and TNF-α production, as well as gene expression of *CCL4*, *IL1B* and *TLR4* in *Mtb-* compared to *Maf*-infected patients. Our study confirms differences in clinical features and immune genes expression and concentration of proteins associated with inflammatory processes between *Mtb*- and *Maf*-infected patients following anti-tuberculosis treatment These findings have public health implications for treatment regimens, and biomarkers for tuberculosis diagnosis and susceptibility.

## Author Summary

In the Gambia tuberculosis is caused my two major lineages within the *Mycobacterium tuberculosis* complex (MTBC), *M*. *tuberculosis* lineages 4 and *M*. *africanum* lineage 6. Our analysis of 26 *M*. *africanum*- and 49 *M*. *tuberculosis* -HIV-negative tuberculosis patients’ clinic parameters and antigen-stimulated blood cytokines concentration and genes expression reveal that heterogeneous response to standard anti-tuberculosis treatment might depend on the diversity of MTBC lineages. Before treatment, the two groups of patients had similar clinical parameters, but greater improvement was observed in *M*. *tuberculosis-* compared to *M*. *africanum* -infected patients post-treatment. This was supported by higher production of inflammation-associated cytokines and genes in unstimulated blood samples from *M*. *africanum*-infected patients compared to those infected with *M*. *tuberculosis*, who instead had higher level of disease resolution cytokines. In contrast, there were lower cytokine responses in antigen-stimulated blood samples of *M*. *africanum*- compared to *M*. *tuberculosis*-infected patients post-treatment indicating a poorly recovered immune profile. Our results suggest that *M*. *africanum* patients respond relatively poorly to the standard anti-tuberculosis treatment or might have a pre-existing defective immune profile; this could explain why they succumb to less virulent mycobacteria.

## Introduction

*Mycobacterium africanum* (*Maf*) is an ancient lineage of the *Mycobacterium tuberculosis* (*Mtb*) Complex (MTBC), mostly found in West Africa where it causes up to half of all tuberculosis (TB) cases [1]. Apart from descriptions of the epidemiological differences between *Maf* and *Mtb* infection in the human population, differences in underlying immune responses, clinical course and outcome of TB therapy have not been described [2]. Other authors have recently attempted to define biomarkers that are able to predict treatment outcome and if validated, these biomarkers could significantly shorten trials of new TB regimens [3–5]. Ultimately, the performance of such biomarkers might be influenced by the infecting mycobacterial lineage. Previous studies that have assessed whether the rate of response to treatment differs between infecting MTBC lineages obtained conflicting results [6–13], but data from our own laboratory and others [6,12,13] indicate that their responses to treatment are heterogeneous. Different MTBC lineages may have been responsible for the heterogeneous response to the shorter TB treatment regimen containing Gatifloxacin recently tested in West Africa [14].

We have previously shown that although the proportion of activated T cells were similar in *Maf*- and *Mtb*-infected patients pre-treatment, they decreased significantly in *Mtb*-infected patients, while those of *Maf*-infected patients were persistently high but consisted of poorly functional T cells post-treatment [15]. In addition, the transcriptomic and metabolic profiles of *Maf*- and *Mtb*-infected patients while similar at baseline significantly differed by lineage post-treatment mainly due to changes in *Mtb*-infected but not in *Maf*-infected patients [13]. These results suggest that intrinsic host factors determine the immune response to TB and/or differential effect of the standard anti-TB treatment on the two lineages.

This study was conducted to investigate the changes in the host immune response and clinical outcomes following treatment in a larger cohort of *Maf*- and *Mtb*-infected tuberculosis patients before, during and after standard anti-TB treatment. Although we found no differences in the clinical parameters measured and found differences in only few cytokines concentration and gene expression profiles between *Maf*- and *Mtb*-infected patients pre-treatment, many of these showed significant differences post-treatment suggesting either intrinsic lineage-specific difference in response to standard anti-TB therapy and/or in the underlying host immunity.

## Methods

### Ethics statement

Ethical approval was obtained from The Joint Gambian Government/Medical Research Council (MRC) Ethics Committee in The Gambia and the London School of Hygiene & Tropical Medicine Ethics Committee. All patients provided written informed consent.

### Study participants

Sputum smear and culture positive TB patients were recruited at the TB Clinic, MRC Unit, Fajara, The Gambia. On recruitment, we recorded clinical symptoms using a questionnaire that included duration of cough, weight lost, night sweats, and fever; routine clinical assessment including anthropometry (weight, height, skinfold thickness (SFK) and body mass index (BMI)), and tuberculin skin test (TST), as previously reported [16]. Sputum was sent for TB smear and culture. The genotypes of the infecting bacilli in sputum were determined by spoligotyping analysis and assessing the presence or absence of lineage defining Large Sequence Polymorphisms (LSP) RD702 and TbD1 as previously described [15,17]. All patients were HIV-negative with no history of previous TB disease and were enrolled before anti-TB treatment. All patients received conventional therapy of 2 months intensive treatment with Isoniazid, Rifampicin, Pyrazinamide, Ethambutol, followed by a second phase of four months with only Isoniazid and Rifampicin (2HRZE/4HR) [18]. They were actively followed-up at 2 and 6 months of treatment, during which chest x-ray (CXR), haematological and sputum smear examination, and anthropometric measurements were done, and heparinized blood samples collected. All patients were confirmed sputum smear negative at the end of the 6 months treatment.

### Whole blood stimulation and multiplex cytokine assays

Undiluted whole blood (180 μL) was incubated overnight (16 hours) in duplicate with 20 μL of medium alone or phytohaemagglutinin (PHA-L, Sigma-Aldrich, UK; 5 μg/ml), purified protein derivative (*Mtb*-PPD; Staten Serum Institute, Denmark; 10 μg/ml), ESAT-6/CFP-10 peptides pool [(EC, ProImmune, UK; 2.5 μg/mL/peptides), EC amino acid sequence is identical in *Maf* and *Mtb* lineages [19], or whole mycobacteria *Mtb* H37Rv and *Maf* GM041182 used both live [final multiplicity of infection (MOI) 1:2 (bacteria: monocytes) and heat-killed (6 x 10^5^ cfu/mL)] [15].

After overnight culture, supernatants were collected from each well, TriReagent (Ambion, Foster City, USA) was added to the pellet, and both were stored at -20°C till analysis. The supernatants were analysed using a Bio-Plex Pro 27-plex kit (cat# M50OKCAFOY, BIO-RAD Laboratories; Belgium) for IL-1β, IL-1RA, IL-2, IL-4, IL-5, IL-6, IL-7, IL-8 (CXCL8), IL-9, IL-10, IL-12p70, IL-13, IL-15, IL-17A, Eotaxin (CCL11), Basic FGF, granulocyte colony-stimulating factor [G-CSF], granulocyte-macrophage CSF [GM-CSF], IFN-γ, IP-10 (CXCL10), MCP-1 (CCL2), MIP-1α (CCL3), MIP-1β (CCL4), PDGF-ββ, RANTES (CCL5), TNF-α, and VEGF following the standard protocol provided by the manufacturers. Plates were immediately read on the Bio-Plex reader using Bio-Plex Manager software (version 4.1.1; Bio-Rad, USA) with five-parameter logistic (5-PL) algorithms and a low PMT setting. All standards were run in duplicate. OOR> and OOR< values were assigned the highest and lowest standard values multiplied or divided by 2 respectively.

### RNA extraction and dual colour Reverse Transcription Multiplex Ligation-dependent Probe Amplification (dcRT-MLPA)

RNA was isolated from stimulated blood pellets lysed in TriReagent (Ambion, Foster City, USA) using a Chloroform/RNeasy (Qiagen, Crawley, UK) protocol following manufacturer’s instructions. Dual colour RT-MLPA was performed as described previously [20,21]. Briefly, 100–150 ng RNA was reverse transcribed using 80nM of target-specific RT primers, 1x MMLV reverse transcriptase and 0.4 mM of each dNTP. cDNA was denatured and hybridized overnight at 60°C with 4 nM of probe mix containing left- and right-hand probes of 85 genes. After ligating the hybridized probes with ligase-65 for 15 min at 54°C, PCR amplification of the ligation products was performed with specific SALSA FAM-labelled MLPA primers, HEX-labelled MAPH primers (1 μL of 2 μM each, forward primer 5’-GGCCGCGGGAATTCGATT-3’ and reverse primer 5’-GCCGCGAATTCACTAGTG-3’), 14.75 μL H_2_0 and 0.25 μL SALSA polymerase. Primers and probes were from Sigma-Aldrich Chemie (Zwijndrecht, The Netherlands) and MLPA SALSA reagents from MRC-Holland (Amsterdam, The Netherlands). Thermal cycling conditions were 33 cycles of 30s at 95°C, 30s at 58°C and 60s at 72°C, followed by 1 cycle of 20min at 72°C. PCR products were diluted 1:10 in HiDi formamide containing 400 HD ROX size standard and analysed on an Applied Biosystems 3730 capillary sequencer in GeneScan mode (Applied Biosystems, Foster City, USA).

Data were analysed using GeneMapper 4.0 software package (Applied Biosystems, Warrington, UK) and peak areas were exported to a Microsoft Excel file for downstream analysis. Data were subsequently normalized to GAPDH housekeeping gene and signals below the threshold value for noise cut-off (peak area #200) were assigned threshold value for noise cut-off. A positive control that encompassed the complement reverse sequence of the combined target-specific sequences of the left and right hand half-probes was used for all runs.

### Statistical analysis

Demographic and clinical characteristics were compared between *Maf*- and *Mtb*-infected patients using Mann-Whitney test for continuous variables and Fisher’s exact test for categorical variables. BMI, skinfold thickness and haematology parameters were logarithmically transformed. No transformation was needed for the chest X-ray score [16], and all were analysed using a random intercept model based on restricted maximum likelihood (REML) estimation.

The cytokine responses were positively skewed and contained zero as values. We first added a constant 0.5 to all cytokine responses as suggested by Yamamura [22], then used a base-2 logarithmic transformation to reduce skewness. Three-level random-intercept model (time points nested in cytokines nested in patients) was fitted to account for the dependence of the cytokine responses within subject and between time points. The model included triple interaction terms between cytokines, lineages, stimulants and time points to estimate the difference in infecting lineages effect on cytokine production in blood incubated with medium alone, as well as the incremental difference in infecting lineages effect induced by each stimulant at each treatment time points. This approach did not require any background subtraction. Contrast analysis was used to estimate differences in infecting lineages effect on cytokine production with Sidak multiple comparison correction [23].

Gene expression data were available for each subject at the same time points as for the cytokines. At each time point, the effect of the four culture conditions (Medium, ESAT-6/CFP-10, live *Maf* and live *Mtb*) on the expression of 85 selected immune-related genes were assessed. Thirty (30) genes showed expression data above the cut-off value (200) for ≤1% of the patients and were discarded from the analysis to avoid inflation of the cut-off value. The remaining 55 genes were log2 transformed and analysed as described for cytokines’ data.

Predicted values of clinical outcomes, cytokines production and genes expression from the constructed model were used to study kinetics following anti-TB treatment for each group and plotted using R software. We tested for interaction between lineages and treatment time points using the Wald test. All analyses were adjusted for age, gender and ethnicity and performed using STATA 12.1 (StataCorp, USA). Statistical significance was considered at p-value ≤0.05.

## Results

### Study participants

**Fig 1:**
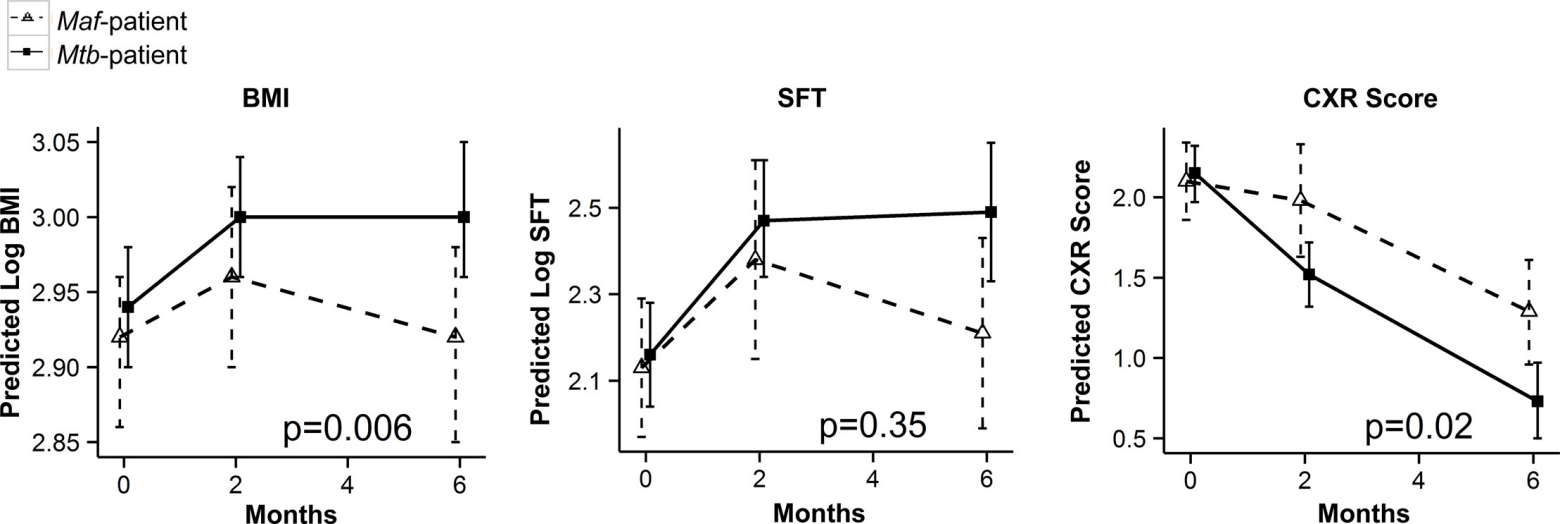
Kinetics of the clinical outcomes in *Maf-* and *Mtb*-infected patients following treatment. Line plots show the predicted mean and its 95% CI of Log transformed Body Mass Index (BMI) and Skinfold Thickness (SFT) in *Maf-* and *Mtb*-infected groups at 0, 2 and 6 months of treatment, Chest X-ray (CXR) was not Log transformed. Wald test through contrasts analysis following a random-intercept model adjusted for age, sex and ethnicity was used to assess interaction between lineage group and time point on clinical response. *Maf* group (dashed lines) and *Mtb* group (solid lines) respectively. P-values of the interactions are shown.

Seventy-five HIV-negative TB patients were enrolled in this study, 26 were infected with *Maf*-lineage 6 and 49 with *Mtb*-lineage 4. Before treatment, *Maf-* and *Mtb*-infected patients had similar clinical symptoms, age, sex, ethnicity, sputum smear microscopy grade and TST results (Table 1). The BMI, skinfold thickness and CXR scores were also similar in both groups at enrolment, but following treatment these were more significantly improved in *Mtb*- compared to *Maf*-infected patients post-treatment after adjusting for age, sex and ethnic group (p = 0.02, p = 0.04 and p = 0.007 respectively; Table 1). The BMI and CXR scores were significantly affected by the infecting lineages following treatment (interaction p = 0.006 and p = 0.02 respectively; Fig 1). Mean corpuscular volume (MCV) was significantly higher in *Mtb-* compared to *Maf*-infected patients before and post-treatment after adjusting for the mentioned potential confounders (p = 0.02 and p = 0.03; respectively), while all other measured haematology parameters were similar between the groups (Table 1). The monocytes/lymphocytes (M:L) ratio was similar before treatment but higher in *Maf*- compared to *Mtb*-infected patients post-treatment after adjusting for confounders (p = 0.05 respectively; Table 1).

**Table 1:** Demographic, clinical and haematology characteristics of *M*. *africanum* and *M*. *tuberculosis* patients.

	*M*. *africanum*		*M*. *tuberculosis*		Unadjusted		Adjusted	
	n	Positive	n	Positive	ED (95%CI)	*P*	AED (95%CI)	*P*
Number of cases at enrolment^a^		26 (35)		49 (65)				
**Age** in years, median (range)	26	30.5 (19–66)	49	27 (15–79)		0.39		
**Sex (%)** (Female)	26	8 (27)	49	20 (38)		0.40		
**Ethnicity** **(%)**	26		49			0.50		
Mandinka		8 (27)		23 (43)				
Jola		10 (33)		5 (9)		0.084		
Fula		4 (13)		10 (19)		0.89		
Wolof		3 (10)		6 (12)		0.79		
Other		5 (17)		9 (17)		0.89		
**Maximum smear grade** **(%)**	26		49			0.18		
1+		5 (19)		3 (6)				
2+		6 (23)		18 (37)		0.066		
3+		15 (58)		28 (57)		0.16		
TST	13	12 (92)	28	24 (89)		0.40		
**BMI, median (range)**								
Enrolment	26	18.6 (13–22)	49	18.6 (13–32)	0.04 (-0.04,0.12)	0.28	0.04 (-0.05,0.13)	0.39
6 months	14	18.6 (17–21)	25	20.2 (17–36)	0.12 (0.04,0.2)	**0.004**	0.11 (0.02,0.20)	**0.02**
**Skinfold thickness median (range) in mm**								
Enrolment	26	8 (3–20)	49	8 (3–20)	0.08 (-0.08,0.25)	0.33	0.03 (-0.17,0.23)	0.76
6 months	14	8.5 (3–20)	27	14 (2–25)	0.2 (0.07,0.4)	**0.006**	0.28 (-0.01,0.55)	**0.04**
**Chest X-Ray (Moderate & Severe Disease)**								
Enrolment	26	21 (81)	48	44 (92)	0.009 (-0.2,0.3)	0.94	0.05 (-0.25,0.35)	0.74
6 months	11	7 (64)	18	2 (11)	-0.55 (-0.8, -0.3)	**0.000**	-0.55 (-0.95, -0.15)	**0.007**
**Haematology parameter median (range)**								
**Haemoglobin (mg/dL)** Enrolment	26	11.5 (8.7–14.6)	43	10.7 (7.5–14.2)	-0.06 (0.13, -003)	0.06	-0.06 (-0.12, 0.01)	0.09
6 months	20	13.4 (11.5–16.8)	30	13.7 (11.3–23.1)	0.01 (-0.06, 0.08)	0.80	0.01 (-0.06, 0.08)	0.74
**WBC (x10**^**9**^**/L)** Enrolment	26	7.2 (3.8–19.4)	43	7.6 (3.6–15.3)	0.1 (-0.06, 0.26)	0.20	0.10 (-0.07, 0.26)	0.24
6 months	20	5.2 (3.2–7.8)	30	4.9 (2.4–8.4)	-0.04 (-0.23, 0.14)	0.63	-0.05 (-0.24, 0.13)	0.56
**Granulocytes (x10**^**9**^**/L)** Enrolment	26	4.3 (1.2–15.8)	43	5.5 (1.4–11.4)	0.21 (-0.07, 0.49)	0.13	0.19 (-0.10, 0.47)	0.20
6 months	20	3 (0.9–13.1)	30	3.4 (1.4–5.7)	-0.02 (-0.33, 0.29)	0.89	-0.03 (-0.34, 0.29)	0.87
**Lymphocytes (x10**^**9**^**/L)** Enrolment	26	1.5 (0.3–8.1)	43	1.7 (0.6–4.2)	0.08 (-0.16, 0.33)	0.50	0.09 (-0.17, 0.34)	0.50
6 months	20	2.8 (0.8–6.3)	30	3.7 (1.6–8.6)	0.03 (-0.25, 0.32)	0.82	0.02 (-0.28, 0.31)	0.91
**Monocytes (x10**^**9**^**/L)** Enrolment	26	0.5 (0.3–1.4)	43	0.5 (0.1–1.2)	0.008 (-0.20, 0.21)	0.93	-0.004 (-0.22, 0.21)	0.97
6 months	20	0.7 (0.3–1.4)	30	0.6 (0.3–1.4)	-0.20 (-0.43, 0.04)	0.1	-0.21 (-0.45, 0.03)	0.08
**M:L ratio** Enrolment	26	0.31 (0.12–1.14)	43	0.29 (0.06–0.87)	-0.06 (-0.30, 0.18)	0.64	-0.06 (-0.30, 0.19)	0.65
6 months	20	0.23 (0.11–0.62)	30	0.18 (0.08–0.45)	-0.27 (-0.55, -0.001)	**0.049**	-0.27 (-0.54, 0.005)	**0.05**
**MCV (fL)** Enrolment	25	77.8(59.2–92.5)	42	79.6 (60.6–92.9)	0.02 (-0.02, 0.07)	0.29	0.05 (0.004, 0.09)	**0.03**
6 months	20	83.7 (67.6–94.8)	30	85.1 (69.3–97.9)	0.04 (-0.01, 0.08)	0.15	0.06 (0.01, 0.10)	**0.02**
**Platelets (x10**^**9**^**/L)** Enrolment	26	376 (131–678)	43	410 (101–663)	0.09 (-0.08, 0.26)	0.30	0.09 (-0.09, 0.26)	0.33
6 months	20	238 (161–355)	30	230 (74–387)	-0.06 (-0.25, 0.13)	0.51	-0.07 (-0.26, 0.13)	0.50

^a^ Total number of patients recruited = 75.

Abbreviations: BMI, body mass index; CXR, chest X-ray; 1+, 2+ & 3+ = Density of Mycobacteria into patient sputum; TST: tuberculin skin test, *Maf* = *M*. *africanum*; *Mtb* = *M*. *tuberculosis;* WBC = white blood cells, MCV = mean corpuscular volume, M:L: monocytes/lymphocytes ratio, ED = Estimated Difference between *Mtb* vs. *Maf*-infected patients, AED = Adjusted ED.

Significant p-values are highlighted in bold.

### *Maf-* and *Mtb*-infected patients’ cytokine response to stimulants differs after treatment

**Fig 2:**
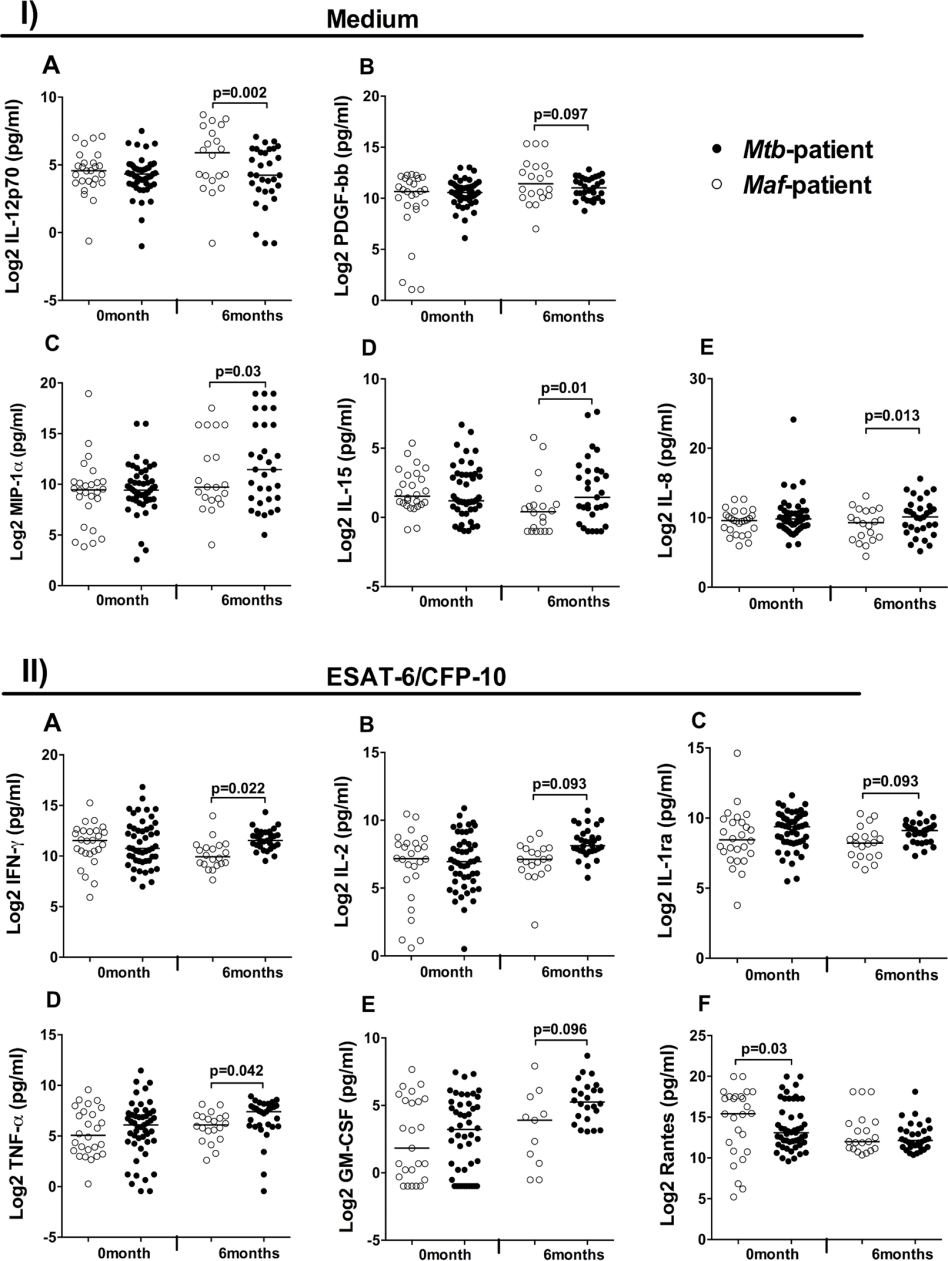
Differential cytokine production between *Maf-* and *Mtb*-infected patients before and after treatment. Whole blood incubated overnight with medium only (I), revealed differences in the concentrations of IL12p70 (A), PDGF-ββ (B), MIP-1α (C), IL-15 (D) and IL-8 (E) between *Mtb-* and *Maf*-infected patients at 6 month of treatment. (II) Only ESAT-6/CFP-10 stimulation induced significant differences in cytokine production between *Mtb-* and *Maf*-infected patients above the background level of IFN-γ (A), IL-2 (B), IL-1ra (C), TNF-α (D), GM-CSF (E) and Rantes (F). Dot plots show log-2 transformed cytokine concentrations measured with Bio-Plex assay. Horizontal bars indicate median cytokine concentration by lineage groups, *Maf*-infected patients (closed circles, n = 26 and 20) and *Mtb*-infected patients (open circles, n = 49 and 31) respectively at 0 and 6 months of TB treatment. Log-2 transformed cytokine concentrations were compared between lineage groups using a random-intercept model adjusted for age, sex and ethnicity, and Sidak multiple comparison correction. Contrasts analysis was used after estimation to compute the difference in each cytokine concentration between lineage groups and used Wald test for assessing the significance at each time point. P-values of the differences are shown.

Before treatment, only stimulation with ESAT-6/CFP-10 induced a significant difference and this was seen only for RANTES (CCL5) production, which was higher in *Maf*- compared to *Mtb*-infected patients (p = 0.03; Fig 2; S1 Table). In contrast, many cytokines showed significantly different responses between *Mtb*- and *Maf*-infected patients post-treatment. In unstimulated blood supernatants, concentrations of IL-8 (p = 0.013), IL-15 (p = 0.01) and MIP-1α (p = 0.027) were significantly higher in *Mtb*- compared to *Maf*-infected patients (Fig 2; S2 Table), whereas IL-12p70 (p = 0.002) and PDGF-β (p = 0.097) were higher in *Maf*- compared to *Mtb*-infected patients. Stimulation with ESAT-6/CFP-10 induced the greatest differences in cytokines production between the two groups. IFN-γ (p = 0.022), TNF-α (p = 0.042), IL-2 (p = 0.093), IL-1ra (p = 0.093), and GM-CSF (p = 0.096) were higher in *Mtb*- compared to *Maf*-infected patients after Sidak multiple comparisons correction (Fig 2; S2 Table).

### Kinetics of specific cytokines production following treatment of *Mtb-* and *Maf*-infected patients

**Fig 3:**
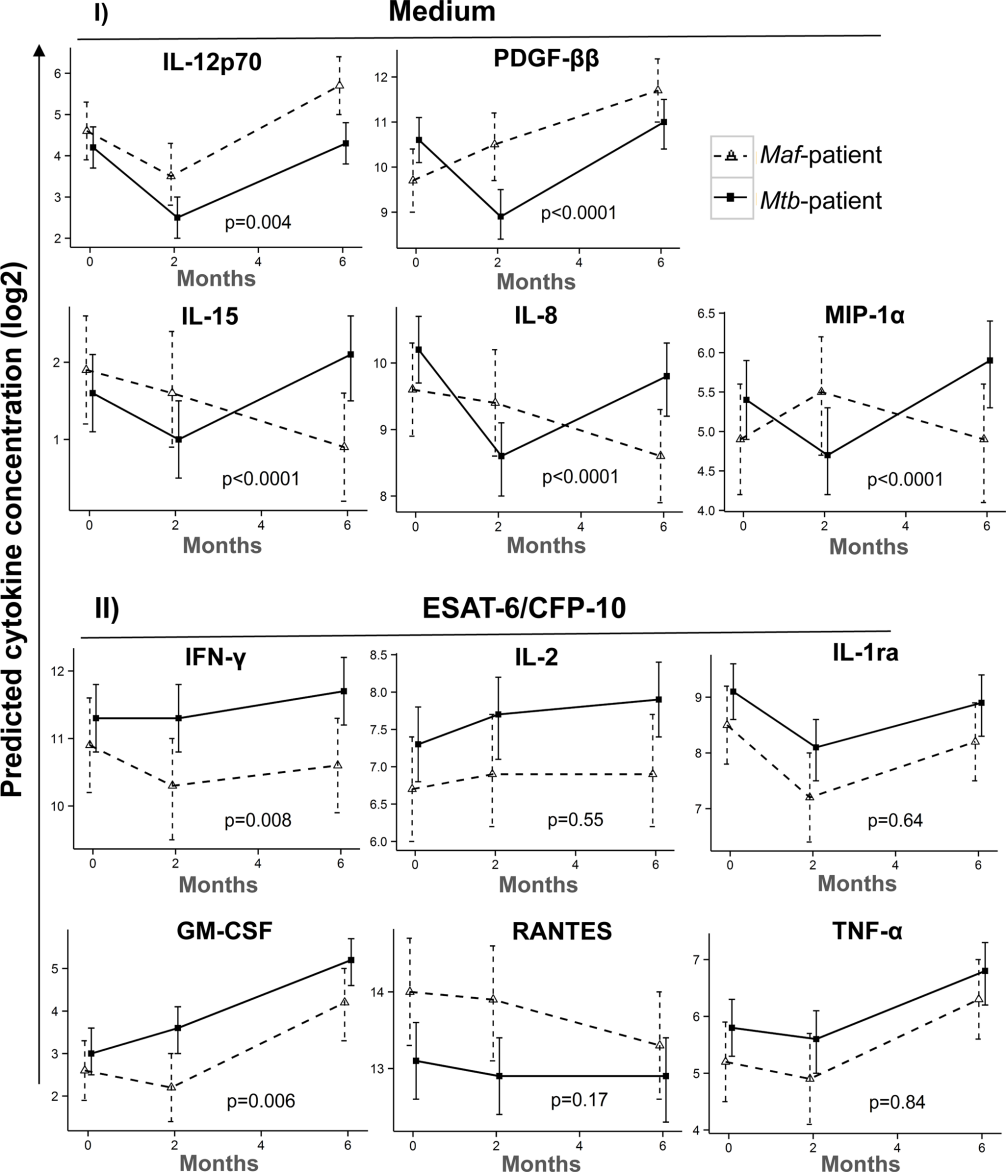
Kinetics of cytokines production between *Maf-* and *Mtb*-infected groups following treatment. The kinetics of cytokine expression showing either strong or weak evidence of difference between lineage groups following overnight incubation with Medium alone (I) or ESAT-6/CFP-10 (II). Line plots show the predicted mean and its 95% confidence interval (95% CI) of log-2 transformed cytokines concentration in *Maf-* and *Mtb*-infected groups at 0, 2 and 6 months of treatment. Wald test through contrasts analysis following a random-intercept model adjusted for age, sex and ethnicity was used to assess interaction between lineage group and time point on cytokine production. The legend shows *Maf*-infected group (dashed lines) and *Mtb*-infected group (solid lines) respectively. P-values of the interactions are shown.

The kinetics of differentially produced cytokines between the groups was assessed in order to understand the effect of treatment on cytokine responses in each group. In the unstimulated samples, the patterns of IL-12p70 and IL-15 were similar at 2 months, but differed significantly at 6 months of treatment, whereas those of IL-8, MIP-1α and PDGF-ββ differed already at 2 months of treatment between the groups (interaction p<0.001; Fig 3A). In response to ESAT-6/CFP-10 stimulation, the patterns of IFN-γ and GM-CSF were significantly different between the groups at 2 months of treatment (interaction p<0.01; Fig 3B).

### Differences in gene expression profiles between *Maf-* and *Mtb*-infected patients following treatment

**Fig 4:**
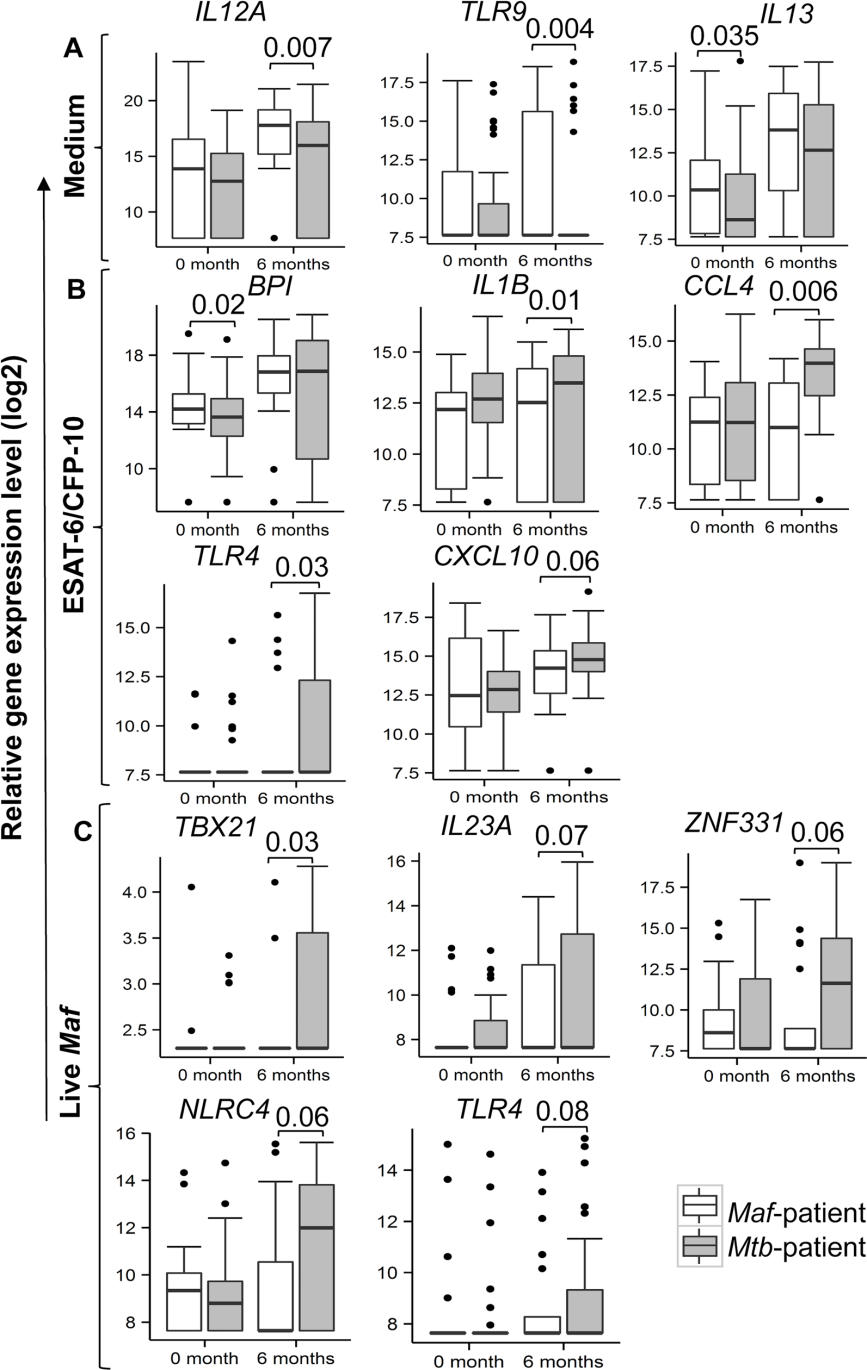
Gene expression profiles differ between *Maf-* and *Mtb*-infected patients before and after treatment. dcRT-MLPA was performed on RNA extracted from whole blood incubated overnight with medium only (A), ESAT-6/CFP-10 (B) and live *Maf* (C). Median gene expression levels (peak areas normalized to GAPDH and log2 transformed) of the indicated genes are shown in box-and-whisker plots. Equal number of samples were analysed at 0 and 6 months of treatment in each group of *Maf*-infected (n = 20; white boxes) and *Mtb*-infected (n = 31; grey boxes) patients, respectively. Log-2 transformed gene expression data were compared between the groups using contrast analysis following a random-intercept model adjusted for age, sex and ethnicity, and Sidak multiple comparison correction. Wald test was used to assess the significance at each time point. P-values of significant differences are shown.

Prior to treatment, there were significantly higher expression of *IL13* in unstimulated (p = 0.035), and *BPI* in ESAT-6/CFP-10 stimulated (p = 0.02) whole blood samples in *Maf-* compared to *Mtb*-infected patients (Fig 4, S3 Table). Likewise, post-treatment, *TLR9* and *IL12A* expression were significantly higher in *Maf-* compared to *Mtb*-infected patients in the unstimulated whole blood (p = 0.004 and p = 0.007 respectively; Fig 4, S4 Table). ESAT-6/CFP-10 stimulation induced significantly higher expression of *IL1B* (p = 0.01), *CCL4* (p = 0.006), *TLR4* (p = 0.03) and *CXCL10* (p = 0.06) in *Mtb-* compared to *Maf*-infected patients. Furthermore, stimulation with live *Maf* induced higher expression of *TBX21* (p = 0.03), *NLRC4* (p = 0.06), *ZNF331* (p = 0.06), *IL23A* (p = 0.07) and *TLR4* (p = 0.08) in *Mtb-* than *Maf*-infected patients (Fig 4, S4 Table).

### Kinetics of gene expression in *Maf-* and *Mtb*-infected patients following treatment

**Fig 5:**
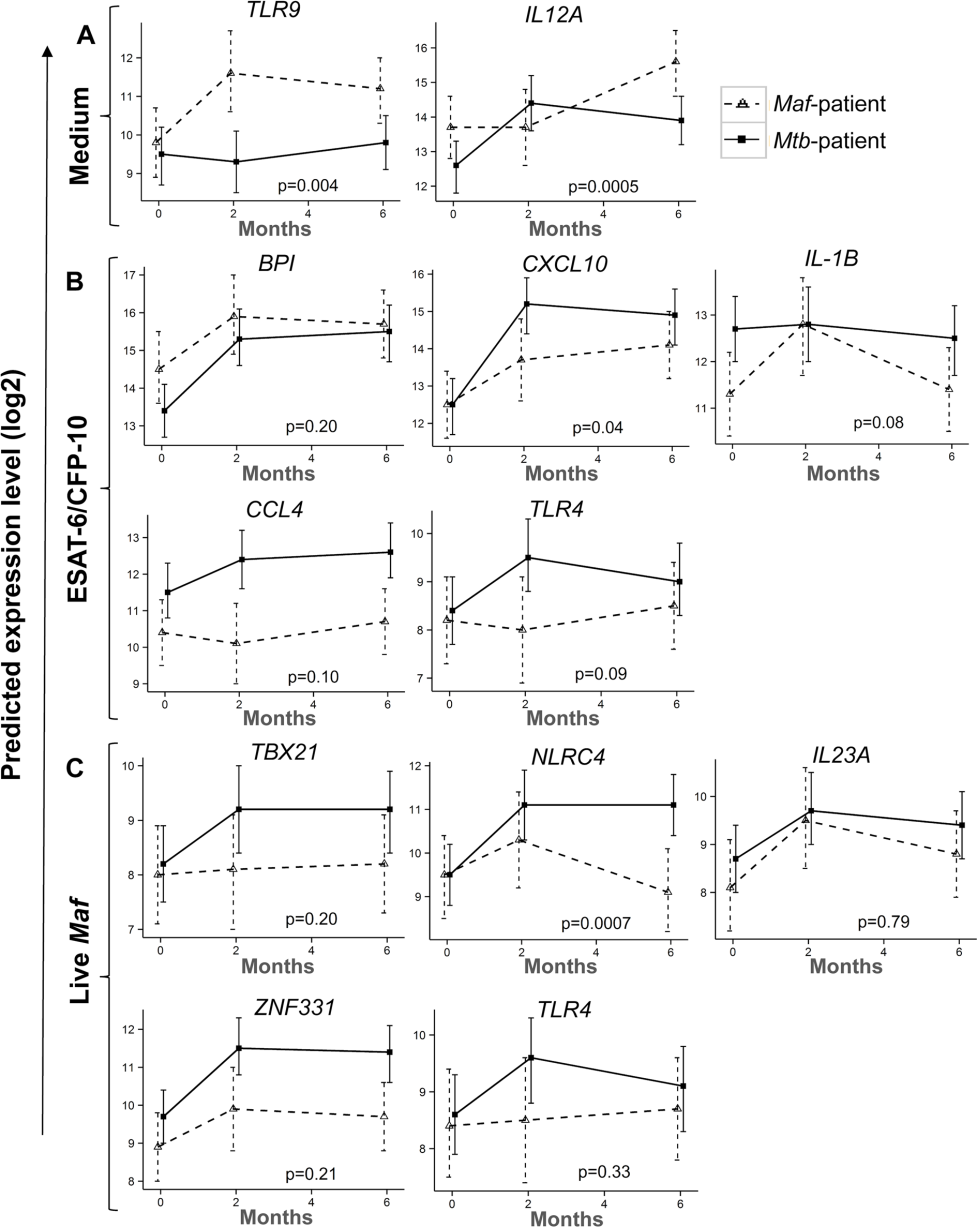
Kinetics of gene expression in *Maf-* and *Mtb*-infected groups following treatment. The expression kinetics of genes that showed significant differences between *Maf-* and *Mtb*-infected groups following overnight incubation with Medium alone (A), ESAT-6/CFP-10 (B) and live *Maf* (C). Line plots show the predicted mean and its 95% CI of log2 transformed gene expression levels in *Maf-* and *Mtb*-infected groups at 0, 2 and 6 months of treatment. Wald test through contrasts analysis following a random-intercept model adjusted for age, sex and ethnicity was used to assess interaction between lineage group and time point on gene expression. The legend shows *Maf-infected* group (dashed lines) and *Mtb-infected* group (solid lines) respectively. P-values of the interactions are shown.

In unstimulated whole blood, the patterns of *TLR9* and *IL12A* expression were significantly different between *Maf* and *Mtb*-infected patients at 2 months of treatment (interaction p = 0.004 and p = 0.0005 respectively; Fig 5A). In ESAT-6/CFP-10 stimulated whole blood, the kinetics of *CXCL10* expression differed between the groups at 2 months of treatment (interaction p = 0.04; Fig 5B), while following live *Maf* stimulation, the kinetics of *NLRC4* expression was significantly different at 2 month of treatment between the groups (interaction p = 0.0007; Fig 5C).

### Interactions of cytokines and genes differentially expressed between *Maf-* and *Mtb*-infected patients

**Fig 6:**
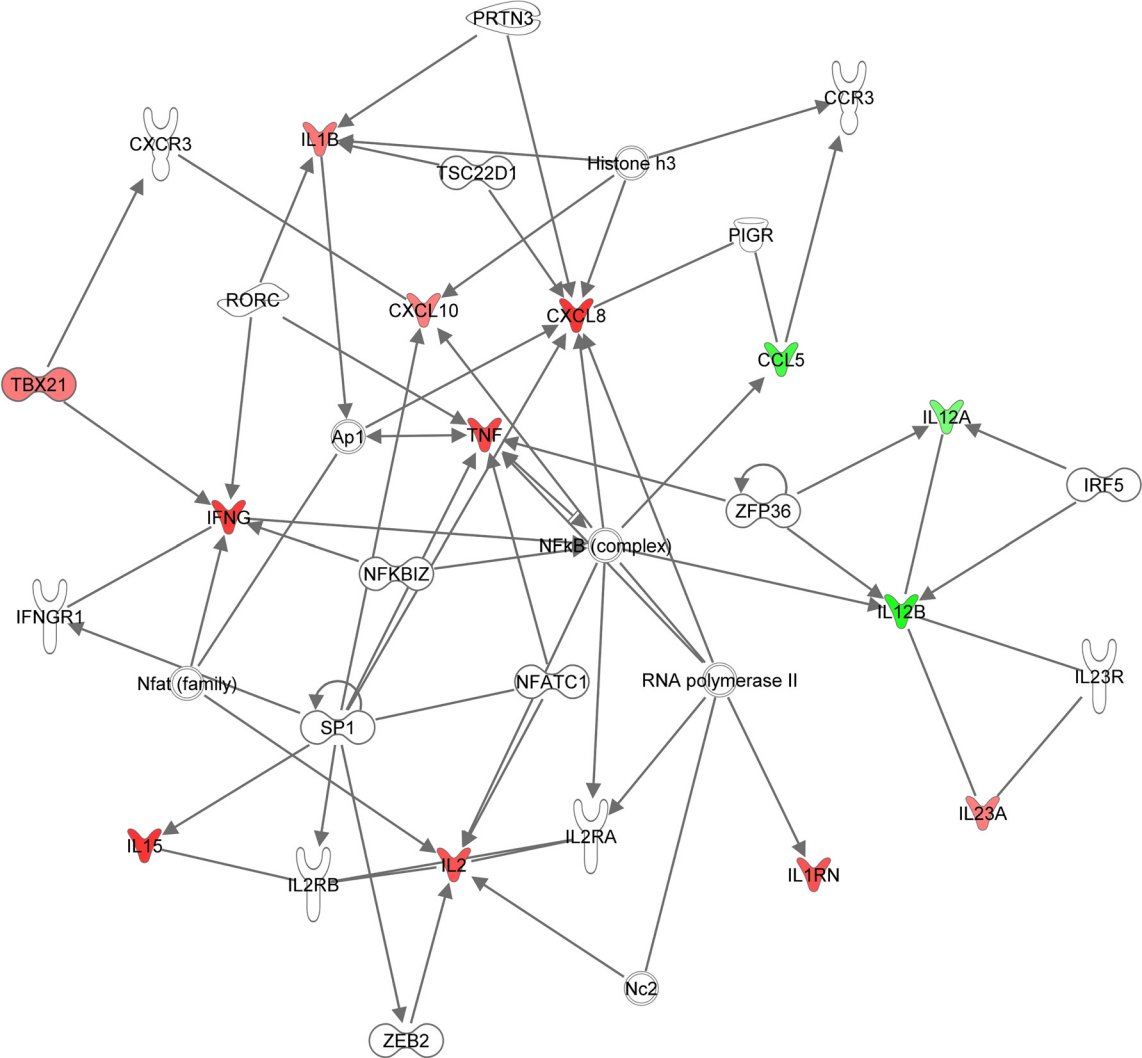
Ingenuity network of direct relationship among genes and cytokines differentially expressed between *Maf-* and *Mtb*-infected patients. Ingenuity network showing 13 of the 23 pro-inflammatory cytokines and genes that were differentially expressed between *Maf-* and *Mtb-* infected patients, centred on NF-κ complex. Genes or cytokines that were higher in *Mtb-* compared to *Maf*- infected patients are depicted in red, those that were lower in green.

The direct relationship among the 23 cytokines and genes differentially expressed between the *Maf* and *Mtb*-infected patients was analysed using the Ingenuity Pathway Analysis (IPA, QIAGEN Redwood City, USA, www.qiagen.com/ingenuity). Ingenuity canonical pathway analysis identified a high enrichment in the communication between innate and adaptive immune cells. Analysis of the upstream regulators revealed a very high enrichment (p = 6.6 x 10^−21^) of the nuclear factor NF-kappa-B p65 subunit (RELA) that regulates more than half (n = 12) of the differentially expressed cytokines and genes between *Maf*- and *Mtb*-infected patients post-treatment. Interestingly, all the cytokines fitted into 2 network functions with the top one containing 13 of the 23 markers and centred on the NFκB complex (Fig 6).

## Discussion

We report here a comparison of clinical and immunological responses before and following standard anti-TB treatment between TB patients infected by *Maf* and *Mtb* lineages of the MTBC in The Gambia. We used an unbiased rigorous statistical approach that accounted for both intra and inter patient’s group variation, experimental condition variability, repeated measurement over time and corrected for multiple comparison to identify biomarkers that are associated with MTBC lineage-specific pathogenesis and response to anti-TB treatment. Overall, before treatment there were no differences in clinical parameters and differences in very few cytokine concentrations and gene expression profiles between *Maf*- and *Mtb*-infected patients. However, after treatment the BMI, CXR, skinfold thickness and immunological responses were more significantly improved in *Mtb-* than in *Maf*-infected patients.

The high similarity in the peripheral blood cells populations, clinical presentation, cytokines production and genes expression profiles before treatment between *Maf*- and *Mtb*-infected patients further support uniformity in host responses after succumbing to infection, irrespective of the infecting lineage of MTBC as previously reported [13,15]. This result also suggests that *Maf* and *Mtb* lineages differences may not affect diagnosis of active TB based on the measurement of soluble cytokines.

The pattern of the changes after treatment are indicative of a quicker recovery from disease in *Mtb*-infected patients, which might be due to their stronger host immunity and/or a better immediate response to treatment of the *Mtb*-lineage 4 [10]. In contrast, the poorer improvement in the clinical outcome following treatment of *Maf*-infected patients could be related to the immune profile. Higher ratio of monocytes/lymphocytes following treatment has been attributed to unresolved infection with on-going inflammation [24]. The high IL-12p70 production in *Maf*-patients correlates with the higher expression of *IL12A* gene, which encodes IL-12p35, a subunit of IL-12p70 produced by macrophages to induce production of inflammatory cytokines from T helper-1 (Th1) and Th17 cells [25,26]. In addition, the increased *TLR9* expression also induces IL-12 production [27] that will further promote prolonged inflammatory processes, which could lead to poor weight gain [28,29] as seen in our *Maf*-infected patients.

IL-15 is a pleiotropic cytokine produced mainly by macrophages, activates a broad range of cells including T and NK cells [30], and promotes the survival of BALB/c mice infected with *Mtb* [31]. Increased MIP-1α levels in unstimulated plasma of HIV-negative TB patients were associated with favourable treatment outcomes [32]. Therefore, the increased IL-15 and MIP-1α in *Mtb*-infected patient after 2 months of treatment could reflect a better response to treatment in contrast to the *Maf*-infected patients.

The significantly higher CCL5 (RANTES) level and *IL13* and *BPI* expression in *Maf*- compared to *Mtb*-infected patients before treatment was not seen post-treatment, implying that treatment did not amplify the differences in the markers already detected pre-treatment but rather revealed new markers. Abundance of *IL13* has predicted progression to active tuberculosis disease in high-risk groups [33], suggesting higher susceptibility as well as a lower ability to mount adequate immune response to recover from disease in our *Maf*-infected patients. This is in line with our previous report of immune exhaustion, and higher prevalence among HIV-positive, severely malnourished and older individuals, of *Maf*-infected patients [1,15,16,34,35], implying that *Maf* mainly causes diseases in a permissive host environment.

ESAT-6/CFP-10 stimulation induced the greatest differences in cytokines concentration and genes expressed between the groups post-treatment. These were mainly pro-inflammatory markers that have previously been associated with protection, such as IFN-γ, and were all significantly higher in *Mtb*- than *Maf*-infected patients. This suggests that the immune system of *Mtb*-infected patients were more capable of mounting a robust response to ESAT-6/CFP-10 on recovering following anti-TB treatment as previously described [15,36,37]. However, other studies have reported the opposite [38,39]. Lower cytokine response post-treatment in *Maf*-infected patients may reflect a poor immune recovery due to an ineffective response to treatment in this group, or a pre-TB immune suppressed state. This is further supported by a generally lower cytokine profile in response to all stimulants in this study, as well as corroborates with the previous finding of increased proportion of HIV-infected amongst patients with *Maf*-caused tuberculosis [34].

The enrichment of NF-κB activation-related genes among the differentially expressed biomarkers between the groups post-treatment is similar to previous reports [40,41]. NF-κB is a key nuclear transcription factor of pro-inflammatory genes activation [41], which corroborates our identified cytokines. We have previously reported high enrichment of HNF4-α, which regulated about 15% of all genes differentially expressed between *Maf*- and *Mtb*-infected patients post-treatment [13]. Low HNF4-α expression has been associated with worst prognosis of hepatocellular carcinoma (HCC) through a robust activation of RELA, whereas higher HNF4-α expression inhibited NF-κB expression and improved HCC outcome [42]. The enrichment of HNF4-α and NF-κB in our datasets is very interesting. Although we cannot yet establish a direct connection based on our current data, this might suggests that the dampened *Maf*-infected patient’s pro-inflammatory response to antigen stimulation post-treatment occurs through the inhibition of NF-κB pathway.

Our study was nested within a platform that has a commendable record of treatment monitoring and completion. [43]. Although both *Mtb* and *Maf*-infected patients had negative smears for AFB by the end of the follow up, differences may not have been observed due to poor sensitivity of the microscopy method used [44]. The more sensitive culture conversion method is not used to monitor treatment in The Gambia and was therefore not used in our study. In addition, although the drug resistance profile of the infecting organisms was not tested, The Gambia has a very low prevalence of TB drug resistance determined in the last survey performed in 2003 [45]. It is therefore unlikely that the different immune profiles observed post-treatment between the groups was due to drug resistance. Moreover, previous studies from West Africa showed that *Maf* is less likely to develop drug resistance compared to *Mtb* [46–48]. However, future studies should include systematic mycobacterial culture to monitor anti-TB treatment response and mycobacterial drug susceptibility testing in order to strengthen this evidence.

There was a striking similarity in the kinetics of cytokines and genes expression between *Mtb*- and *Maf*-infected patients during the first two months, but significant differences at 6 months of treatment. These together with the clinical responses provide a coherent and robust evidence of differential responses to treatment in *Maf*- and *Mtb*-infected patients. This pattern may indicate that the interval for differences to emerge between the groups by 2 months was short and the difference at 6 month of treatment may reflect a slower rate of clinical and immunological disease resolution of *Maf*-infected TB patients. The fact that most differences between the two groups only emerge after 2 months of treatment suggest that *Maf*-infected patient may require longer intensive treatment phase, a longer treatment regimen overall, or reveals the pre-TB disease immune profile of the individuals. These results suggest that *Maf* leaves a permissive host profiles after treatment with poor lung function and health quality in general, which might promotes the susceptibility to future disease. It becomes important to investigate *Maf*-infected patients long-term recovery post-treatment and their risk of relapse or re-infection as a permissive host immunity could favour TB disease from *Maf*, which remains a less virulent bacillus [49] but yet has not been outcompeted by other *Mtb* lineages according to recent report in West Africa [35,48,50–56]. Moreover, *Maf* causes up to half of all tuberculosis cases in West Africa where the same treatment regimen is given irrespective of the infecting MTBC lineage. New host-directed therapeutic approaches that aim to reduce inflammatory responses associated with immunopathology might be proven useful for these patients [57,58]. Furthermore, these results demonstrate that the identification and evaluation of immunological biomarkers to monitor anti-tuberculosis treatment response in West Africa should consider the diversity of MTBC lineages.

Studies in *Maf* non-endemic regions have shown heterogeneous response to TB treatment depending on the infecting MTBC lineages [6,10,59]. However, other studies in highly heterogeneous populations found no effect of MTBC lineages, but rather patient ethnicity was a significant determinant for difference in treatment response [9,11]. Clearly, host and bacteria factors are important determinants for treatment response, therefore stratified approaches to TB treatment that account for MTBC lineages is required to improve treatment outcome especially now with clinical trials of shorter drug treatment regimen for TB [14,60].

In conclusion, our data show differences in clinical parameters and immune genes and proteins associated with inflammatory processes recovery between *Mtb*- and *Maf*-infected patients following anti-tuberculosis treatment. This profile may be an indication of differences in the resolution of disease or the pre-tuberculosis status of the host immune system. These findings may have public health relevance for therapeutic and biomarker discovery purposes, and warrant further investigation for the use of the identified biomarkers as potential targets for preventive or therapeutic intervention against tuberculosis.

## Supporting Information

S1 Checklist STROBE checklist. List of pages and paragraphs containing keys information about this study. The pages and paragraphs numbers responding to the specific questions of the STROBE Checklist are provided to ease the reading of this manuscript and understanding the study.
(DOC)

S1 Table Estimated Difference (ED) and Estimated Incremental Difference (EID) of cytokines production between *Mtb* and *Maf*-infected patients by stimulants, adjusted for age, gender and ethnicity before anti-TB treatment. Show the Estimated Difference (ED) of cytokines production in blood incubated with Medium only and the Estimated Incremental Difference (EID) of cytokines production above the baseline induced by the respective stimulants between *Mtb* and *Maf*-infected patients before treatment. The statistical analyses were done using a random intercept model based on restricted maximum likelihood (REML) and adjusted for age, gender and ethnicity as well as applying Sidak multiple comparison correction. Statistical significant ED and EID are highlighted in bold.
(DOCX)

S2 Table Estimated Difference (ED) and Estimated Incremental Difference (EID) of cytokines production between *Mtb* and *Maf*-infected patients by stimulants, adjusted for age, gender and ethnicity at 6 months of anti-TB treatment. Show the Estimated Difference (ED) of cytokines production in blood incubated with Medium only and the Estimated Incremental Difference (EID) of cytokines production above the baseline induced by the respective stimulants between *Mtb* and *Maf*-infected patients at 6 months of treatment. The statistical analyses were done using a random intercept model based on restricted maximum likelihood (REML) and adjusted for age, gender and ethnicity as well as applying Sidak multiple comparison correction. Statistical significant ED and EID are highlighted in bold.
(DOCX)

S3 Table Estimated Difference (ED) and Estimated Incremental Difference (EID) in genes expression between *Mtb* and *Maf*-infected patients by stimulants, adjusted for age, gender and ethnicity before anti-TB treatment. Show the Estimated Difference (ED) of genes expression in blood incubated with Medium only and the Estimated Incremental Difference (EID) of genes expression above the baseline induced by the respective stimulants between *Mtb* and *Maf*-infected patients before treatment. The statistical analyses were done using a random intercept model based on restricted maximum likelihood (REML) and adjusted for age, gender and ethnicity as well as applying Sidak multiple comparison correction. Statistical significant ED and EID are highlighted in bold.
(XLSX)

S4 Table Estimated Difference (ED) and Estimated Incremental Difference (EID) in genes expression between *Mtb* and *Maf*-infected patients by stimulants, adjusted for age, gender and ethnicity at 6 months of anti-TB treatment. Show the Estimated Difference (ED) of genes expression in blood incubated with Medium only and the Estimated Incremental Difference (EID) of genes expression above the baseline induced by the respective stimulants between *Mtb* and *Maf*-infected patients at 6 months of treatment. The statistical analyses were done using a random intercept model based on restricted maximum likelihood (REML) and adjusted for age, gender and ethnicity as well as applying Sidak multiple comparison correction. Statistical significant ED and EID are highlighted in bold.
(XLSX)
