# Supplementary material for: Host Immune Responses Differ between M. africanum- and M. tuberculosis-Infected Patients following Standard Anti-tuberculosis Treatment
Source: PLoS Negl Trop Dis. 2016 May 18;10(5):e0004701. doi: 10.1371/journal.pntd.0004701 (PMC4871581; doi:10.1371/journal.pntd.0004701)
Supplement: S1 Checklist — List of pages and paragraphs containing keys information about this study. The pages and paragraphs numbers responding to the specific questions of the STROBE Checklist are provided to ease the reading of this manuscript and understanding the study. (DOC) [file pntd.0004701.s001.doc]

STROBE Statement—Checklist of items that should be included in reports of ***cohort studies***

|  | Item No | Recommendation |
| --- | --- | --- |
| **Title and abstract** | 1 | (*a*) Indicate the study’s design with a commonly used term in the title or the abstract  “Differences” “anti-tuberculosis” “treatment” “M. africanum” “M. tuberculosis” |
|  |  | (*b*) Provide in the abstract an informative and balanced summary of what was done and what was found  Page#2 |
| Introduction | | |
| Background/rationale | 2 | Explain the scientific background and rationale for the investigation being reported  Page#4 paragraph#1 & 2 |
| Objectives | 3 | State specific objectives, including any prespecified hypotheses  Page#4 paragraph#3 |
| Methods | | |
| Study design | 4 | Present key elements of study design early in the paper |
| Setting | 5 | Describe the setting, locations, and relevant dates, including periods of recruitment, exposure, follow-up, and data collection  Page#5 paragraph#3 |
| Participants | 6 | 1. Give the eligibility criteria, and the sources and methods of selection of participants. Describe methods of follow-up   Page#6 paragraph#1 |
|  |  | (*b*) For matched studies, give matching criteria and number of exposed and unexposed |
| Variables | 7 | Clearly define all outcomes, exposures, predictors, potential confounders, and effect modifiers. Give diagnostic criteria, if applicable  Page#5 paragraph#3 |
| Data sources/ measurement | 8* | For each variable of interest, give sources of data and details of methods of assessment (measurement). Describe comparability of assessment methods if there is more than one group  Page#5 & 7 |
| Bias | 9 | Describe any efforts to address potential sources of bias |
| Study size | 10 | Explain how the study size was arrived at  Page#9 paragraph#1, Page#4 paragraph#3 |
| Quantitative variables | 11 | Explain how quantitative variables were handled in the analyses. If applicable, describe which groupings were chosen and why.  Page#8 & 9 |
| Statistical methods | 12 | 1. Describe all statistical methods, including those used to control for confounding   Page#8 paragraph#3 and Page#9 paragraph#2 |
|  |  | 1. Describe any methods used to examine subgroups and interactions   Page#9 paragraph#2 |
|  |  | 1. Explain how missing data were addressed   Page#8 paragraph#3 |
|  |  | (*d*) If applicable, explain how loss to follow-up was addressed  Page#8 paragraph#3 |
|  |  | (*e*) Describe any sensitivity analyses  N/A |
| Results | | |
| Participants | 13* | 1. Report numbers of individuals at each stage of study—eg numbers potentially eligible, examined for eligibility, confirmed eligible, included in the study, completing follow-up, and analysed   Page#25 & 26 Table1. |
|  |  | (b) Give reasons for non-participation at each stage |
|  |  | (c) Consider use of a flow diagram |
| Descriptive data | 14* | 1. Give characteristics of study participants (eg demographic, clinical, social) and information on exposures and potential confounders   Page#9 paragraph#3 & page#10 paragraph#1 |
|  |  | 1. Indicate number of participants with missing data for each variable of interest   Page#25 & 26 Table1. |
|  |  | 1. Summarise follow-up time (eg, average and total amount)   Page#25 & 26 Table1. |
| Outcome data | 15* | Report numbers of outcome events or summary measures over time  Page#25 & 26 Table1, Supporting information Table 1, 2, 3 & 4. |
| Main results | 16 | 1. Give unadjusted estimates and, if applicable, confounder-adjusted estimates and their precision (eg, 95% confidence interval). Make clear which confounders were adjusted for and why they were included   Page#9 paragraph# 2 & 3 |
|  |  | 1. Report category boundaries when continuous variables were categorized   Page#25 & 26. Table 1 |
|  |  | (*c*) If relevant, consider translating estimates of relative risk into absolute risk for a meaningful time period  N/A |
| Other analyses | 17 | Report other analyses done—eg analyses of subgroups and interactions, and sensitivity analyses  Page#12 paragraph#2 |
| Discussion | | |
| Key results | 18 | Summarise key results with reference to study objectives  Page#13 Paragraph#1 |
| Limitations | 19 | Discuss limitations of the study, taking into account sources of potential bias or imprecision. Discuss both direction and magnitude of any potential bias  Page#16 paragraph#2 |
| Interpretation | 20 | Give a cautious overall interpretation of results considering objectives, limitations, multiplicity of analyses, results from similar studies, and other relevant evidence  Page#13 - 18 |
| Generalisability | 21 | Discuss the generalisability (external validity) of the study results  Page#17 paragraph#2 |
| Other information | | |
| Funding | 22 | Give the source of funding and the role of the funders for the present study and, if applicable, for the original study on which the present article is based  Provided in the submission portal. |

*Give information separately for exposed and unexposed groups.

**Note:** An Explanation and Elaboration article discusses each checklist item and gives methodological background and published examples of transparent reporting. The STROBE checklist is best used in conjunction with this article (freely available on the Web sites of PLoS Medicine at http://www.plosmedicine.org/, Annals of Internal Medicine at http://www.annals.org/, and Epidemiology at http://www.epidem.com/). Information on the STROBE Initiative is available at http://www.strobe-statement.org.
