# Supplementary material for: Host Immune Responses Differ between M. africanum- and M. tuberculosis-Infected Patients following Standard Anti-tuberculosis Treatment
Source: PLoS Negl Trop Dis. 2016 May 18;10(5):e0004701. doi: 10.1371/journal.pntd.0004701 (PMC4871581; doi:10.1371/journal.pntd.0004701)
Supplement: S1 Table — Show the Estimated Difference (ED) of cytokines production in blood incubated with Medium only and the Estimated Incremental Difference (EID) of cytokines production above the baseline induced by the respective stimulants between Mtb and Maf-infected patients before treatment. The statistical analyses were done using a random intercept model based on restricted maximum likelihood (REML) and adjusted for age, gender and ethnicity as well as applying Sidak multiple comparison correction. Statistical significant ED and EID are highlighted in bold. (DOCX) [file pntd.0004701.s002.docx]

**S1. Table: Estimated Difference (ED) and Estimated Incremental Difference (EID) strain effect on the log response between *Mtb* and *Maf* patients by stimulant and cytokine, adjusted for age, gender and ethnicity before anti-TB treatment.**

|  | **Stimulant** | | | | | | | | **P^#^** |
| --- | --- | --- | --- | --- | --- | --- | --- | --- | --- |
|  | Medium | EC | Live Maf | Live Mtb | Killed Maf | Killed Mtb | PHA | PPD |  |
| Cytokines | ED (95%CI) | EID (95%CI) | EID (95%CI) | EID (95%CI) | EID (95%CI) | EID (95%CI) | EID (95%CI) | EID (95%CI) |  |
| IFN-γ | -0.44 (-1.30;0.43) | 0.80 (-0.14;1.73) | -0.08 (-0.86;0.70) | 0.11 (-0.67;0.89) | -0.13 (-0.91;0.65) | 0.04 (-0.75;0.82) | 0.53 (-0.26;1.32) | 0.01 (-0.77;0.80) | 0.17 |
| IL-1β | 0.18 (-0.68;1.05) | 0.46 (-0.32;1.24) | -0.69 (-1.64;0.191) | -0.44 (-1.22;0.34) | -0.13 (-0.91;0.65) | -0.24 (-1.02;0.54) | -0.05 (-0.83;0.74) | -0.35 (-1.13;0.44) | 0.13 |
| IL-1RA | 0.05 (-0.82;0.91) | 0.56 (-0.22;1.34) | -0.14 (-0.92;0.65) | 0.13 (-0.65;0.91) | -0.13 (-0.91;0.65) | 0.10 (-0.68;0.88) | 0.34 (-0.45;1.13) | 0.08 (-0.70;0.87) | 0.59 |
| IL-2 | 0.10 (-0.76;0.97) | 0.56 (-0.22;1.34) | -0.07 (-0.85;0.71) | -0.001 (-0.78;0.78) | -0.08 (-0.86;0.71) | 0.03 (-0.75;0.82) | 0.20 (-0.58;0.99) | 0.10 (-0.69;0.88) | 0.71 |
| IL-4 | -0.01 (-0.88;0.86) | 0.09 (-0.69;0.87) | -0.05 (-0.84;0.73) | -0.02 (-0.80;0.77) | -0.06 (-0.84;0.72) | 0.01 (-0.77;0.79) | 0.14 (-0.65;0.93) | -0.06 (-0.84;0.73) | 0.99 |
| IL-5 | 0.07 (-0.80;0.94) | 0.32 (-0.46;1.10) | -0.08 (-0.86;0.70) | -0.14 (-0.92;0.64) | -0.12 (-0.90;0.66) | 0.08 (-0.70;0.86) | -0.16 (-0.95;0.62) | -0.12 (-0.90;0.66) | 0.89 |
| IL-6 | -0.59 (-1.45;0.28) | 0.53 (-0.25;1.31) | 0.02 (-0.76;0.80) | -0.14 (-0.92;0.64) | -0.03 (-0.82;0.75) | 0.22 (-0.56;1.00) | 0.09 (-0.70;0.88) | 0.08 (-0.70;0.86) | 0.74 |
| IL-7 | 0.03 (-0.84;0.89) | -0.04 (-0.82;0.74) | -0.16 (-0.94;0.62) | -0.22 (-1.00;0.56) | -0.03 (-0.82;0.75) | 0.01 (-0.77;0.79) | 0.05 (-0.74;0.84) | -0.13 (-0.91;0.66) | 0.99 |
| IL-8 | 0.61 (-0.26;1.48) | 0.22 (-0.56;1.00) | -0.27 (-1.05;0.51) | -0.56 (-1.34;0.23) | -0.27 (-1.05;0.52) | -0.17 (-0.95;0.61) | -0.38 (-1.17;0.41) | -0.41 (-1.19;0.37) | 0.60 |
| IL-9 | -0.10 (-0.96;0.77) | -0.07 (-0.85;0.71) | -0.06 (-0.85;0.72) | -0.10 (-0.88;0.69) | -0.05 (-0.83;0.74) | 0.11 (-0.67;0.90) | 0.17 (-0.62;0.96) | -0.18 (-0.96;0.60) | 0.98 |
| IL-10 | 0.01 (-0.86;0.87) | -0.25 (-1.04;0.53) | 0.14 (-0.64;0.93) | -0.01 (-0.79;0.77) | -0.02 (-0.80;0.76) | 0.25 (-0.53;1.03) | 0.51 (-0.27;1.30) | 0.09 (-0.69;0.88) | 0.64 |
| IL-12p70 | -0.36 (-1.23;0.50) | 0.11 (-0.67;0.89) | 0.02 (-0.77;0.80) | -0.07 (-0.85;0.71) | -0.11 (-0.89;0.67) | 0.11 (-0.67;0.90) | 0.12 (-0.67;0.91) | -0.33 (-1.11;0.46) | 0.92 |
| IL-13 | -0.19 (-1.06;0.68) | 0.18 (-0.60;0.97) | 0.01 (-0.77;0.79) | -0.14 (-0.92;0.64) | -0.03 (-0.81;0.75) | 0.11 (-0.68;0.88) | 0.17 (-0.62;0.95) | -0.22 (-1.00;0.56) | 0.94 |
| IL-15 | -0.26 (-1.12;0.61) | -0.001 (-0.78;0.78) | -0.18 (-0.96;0.61) | -0.09 (-0.88;0.69) | -0.05 (-0.83;0.74) | 0.12 (-0.66;0.90) | 0.13 (-0.66;0.91) | 0.15 (-0.63;0.93) | 0.98 |
| IL-17 | 0.44 (-0.43;1.30) | -0.06 (-0.84;0.72) | -0.56 (-1.34;0.22) | -0.65 (-1.43;0.13) | -0.25 (-1.03;0.53) | -0.15 (-0.93;0.63) | 0.02 (-0.77;0.81) | -0.64 (-1.42;0.14) | 0.42 |
| EOTAXIN | -0.53 (-1.40;0.34) | 0.18 (-0.60;0.97) | -0.14 (-0.93;0.64) | -0.03 (-0.82;0.75) | -0.10 (-0.88;0.68) | 0.07 (-0.71;0.86) | 0.02 (-0.77;0.80) | -0.13 (-0.91;0.65) | 0.98 |
| FGF-b | 0.13 (-0.74;1.00) | -0.28 (-1.07;0.50) | -0.33 (-1.11;0.45) | -0.26 (-1.04;0.53) | -0.16 (-0.94;0.63) | -0.25 (-1.03;0.54) | -0.52 (-1.31;0.27) | -0.32 (-1.10;0.47) | 0.99 |
| G-CSF | -0.29 (-1.16;0.58) | 0.14 (-0.64;0.92) | -0.13 (-0.92;0.65) | 0.01 (-0.77;0.79) | -0.05 (-0.83;0.73) | -0.08 (-0.87;0.70) | 0.22 (-0.57;1.01) | 0.05 (-0.73;0.83) | 0.98 |
| GM-CSF | -0.21 (-1.10;0.69) | 0.63 (-0.21;1.46) | -0.18 (-1.02;0.66) | 0.30 (-0.54;1.14) | -0.10 (-0.94;0.74) | -0.04 (-0.88;0.80) | 0.002 (-0.84;0.85) | 0.06 (-0.78;0.90) | 0.53 |
| IP-10 | -0.22 (-1.09;0.65) | 0.42 (-0.36;1.20) | 0.02 (-0.77;0.80) | -0.03 (-0.81;0.75) | -0.13 (-0.92;0.65) | 0.15 (-0.63;0.93) | 0.39 (-0.40;1.18) | -0.11 (-0.89;0.67) | 0.69 |
| MCP-1 | 0.06 (-0.80;0.93) | 0.11 (-0.67;0.89) | 0.01 (-0.78;0.79) | 0.15 (-0.63;0.93) | -0.17 (-0.95;0.61) | 0.21 (-0.57;0.99) | 0.19 (-0.60;0.98) | 0.26 (-0.52;1.04) | 0.95 |
| MIP-1α | 0.55 (-0.31;1.42) | 0.36 (-0.42;1.14) | -0.03 (-0.81;0.75) | 0.04 (-0.75;0.82) | -0.25 (-1.03;0.54) | 0.16 (-0.62;0.95) | -0.17 (-0.96;0.62) | 0.003 (-0.78;0.79) | 0.80 |
| MIP-1β | 0.16 (-0.71;1.02) | 0.32 (-0.46;1.10) | 0.03 (-0.75;0.81) | 0.29 (-0.49;1.07) | -0.08 (-0.86;0.71) | 0.24 (-0.54;1.02) | 0.24 (-0.55;1.03) | -0.24 (-1.03;0.54) | 0.75 |
| PDGF-β | 0.94 (0.08;1.81) | -0.49 (-1.28;0.29) | -0.27 (-1.05;0.51) | -0.46 (-1.24;0.32) | -0.22 (-1.00;0.56) | -0.20 (0.62;0.95) | -0.18 (-0.97;0.61) | -0.36 (-1.14;0.42) | 0.98 |
| RANTES | 0.20 (-0.67;1.06) | **-1.02 (-1.96;-0.08)**** | -0.20 (-0.98;0.58) | -0.23 (-1.01;0.55) | -0.17 (-0.95;0.62) | -0.44 (-1.22;0.35) | -0.58 (-1.37;0.21) | -0.32 (-1.10;0.46) | 0.33 |
| TNF-α | -0.18 (-1.04;0.69) | 0.70 (1.67;1.81) | -0.25 (-1.03;0.54) | 0.40 (-0.38;1.19) | 0.04 (-0.75;0.82) | 0.07 (-0.71;0.85) | 0.13 (-0.65;0.92) | 0.04 (-0.74;0.82) | 0.32 |
| VEGF | -0.46 (-1.32;0.41) | -0.56 (-1.34;0.22) | -0.10 (-0.89;0.68) | -0.44 (-1.22;0.34) | -0.12 (-0.90;0.66) | 0.03 (-0.75;0.81) | -0.06 (-0.85;0.73) | -0.46 (-1.25;0.32) | 0.66 |

^#^: Wald test assessing whether there is evidence of significant differences in the incremental MTBC lineage effect on the corresponding cytokines production between stimulants.

*: Statistically significant at p<0.1.

**: Statistically significant at p<0.05.

***: Statistically significant at p<0.01.

ED: Estimated Difference of cytokines production between *Mtb* vs *Maf*-infected patients in blood incubated with medium alone.

EID: Estimated Incremental Difference of cytokines production between *Mtb* vs *Maf*-infected patients induced by the respective stimulants.

Number highlighted in bold showed statistical significant differences.
